# Carbonaceous Shape-Stabilized Octadecane/Multi-Walled Carbon Nanotube Composite Materials for Enhanced Energy Storage and Electromagnetic Interference Shielding

**DOI:** 10.3390/molecules29184363

**Published:** 2024-09-13

**Authors:** Maria Baikousi, Christina Gioti, Konstantinos C. Vasilopoulos, Argyri Drymiskianaki, Vassilis M. Papadakis, Zacharias Viskadourakis, Angelos Ntaflos, Dimitrios Moschovas, Alkiviadis S. Paipetis, George Kenanakis, Michael A. Karakassides

**Affiliations:** 1 Department of Materials Science and Engineering, University of Ioannina, GR-45110 Ioannina, Greece; christina.a.gioti@gmail.com (C.G.); kovasil@auth.gr (K.C.V.); a.ntaflos@uoi.gr (A.N.); dmoschov@uoi.gr (D.M.); paipetis@uoi.gr (A.S.P.); 2 Institute of Electronic Structure and Laser, Foundation for Research and Technology-Hellas, N. Plastira 100, Vasilika Vouton, GR-70013 Heraklion, Greece; adrym@materials.uoc.gr (A.D.); v.papadakis@uniwa.gr (V.M.P.); zach@iesl.forth.gr (Z.V.); gkenanak@iesl.forth.gr (G.K.); 3 Department of Materials Science and Engineering, University of Crete, GR-70013 Heraklion, Greece; 4 Department of Industrial Design and Production Engineering, University of West Attica, GR-12243 Athens, Greece

**Keywords:** activated carbon, carbon foam, expanded graphite, multi-walled carbon nanotubes (MWCNTs), phase change materials, octadecane, thermal storage, EMI shielding effectiveness

## Abstract

Developing materials for efficient energy storage and effective electromagnetic interference (EMI) shielding is crucial in modern technology. This study explores the synthesis and characterization of carbonaceous shape-stabilized octadecane/MWCNT (multi-walled carbon nanotube) composites, utilizing activated carbon, expanded graphite or ceramic carbon foam, as shape stabilizers for phase change materials (PCMs) to enhance thermal energy storage and EMI shielding, for energy-efficient and advanced applications. The integration of octadecane, a phase change material (PCM) with carbonaceous stabilizers ensures the material’s stability during phase transitions, while MWCNTs contribute to improved thermal storage properties and EMI shielding capabilities. The research aims to develop novel composites with dual functionality for thermal storage and EMI shielding, emphasizing the role of carbon matrices and their MWCNT composites. SEM and CT microtomography analyses reveal variations in MWCNT incorporation across the matrices, influenced by surface properties and porosity. Leaching tests, infrared spectroscopy (FT-IR) and differential scanning calorimetry (DSC) confirm the composite’s stability and high latent heat storage. The presence of nanotubes enhances the thermal properties of octadecane and ΔH values almost reached their theoretical values. EMI shielding effectiveness measurements indicate that the composites show improved electric properties in the presence of MWCNTs.

## 1. Introduction

The demand for efficient energy storage and effective electromagnetic interference (EMI) shielding materials has significantly increased with climate change and the rapid development of technology on electronic devices and systems. Nowadays, a significant challenge is to reduce the cooling and heating demands of buildings and the practical solution is finding new materials that can improve these properties of the building components [1]. Moreover, the need for buildings capable of mitigating the effects of electromagnetic waves has become increasingly critical in our technology-infused surroundings. The swift advancement of electronic devices has heightened concerns about electromagnetic radiation, as it can interfere with device performance and potentially affect human health [2]. In response to these challenges, the design of advanced materials with multifunctional properties has emerged as a promising strategy for achieving diverse and effective solutions in building constructions.

Phase change materials (PCMs) such as paraffins have gained significant attention in the construction industry for their potential applications in energy storage and thermal management systems. PCMs can improve the thermal performance of buildings when integrated into various construction components, such as concrete, gypsum, or ceramic bricks [3]. By incorporating PCMs into these materials, it is possible to mitigate rapid fluctuations in indoor temperatures, thereby decreasing energy losses and lowering heating and cooling requirements without compromising comfort levels. However, these materials are still limited for practical applications due to some drawbacks related to their leakage during the phase transition and low thermal conductivity [4]. Carbonaceous composites, which are also conductive materials, act as effective shape-stabilized materials for PCMs (SS-PCMs) leading to effective thermal storage and parallel EMI shielding systems [5,6,7].

Paraffin octadecane, a well-known PCM with a melting point of 28 °C and high latent heat storage capacity, is an ideal component for thermal energy storage applications [8]. However, its leakage during the phase transition and low thermal conductivity limits its practical applications. To address this issue, researchers have developed composite materials that incorporate octadecane within a carbonaceous matrix, thereby stabilizing its shape and enhancing its thermal and mechanical properties. Such materials encase the PCM, enabling it to remain in its molten state without movement even at high temperatures [9,10]. Some carbonaceous materials, that were used for the effective stabilization of PCMs, are activated carbons, carbon foams, graphite, graphene, single-wall nanotubes, multi-walled nanotubes and carbon nanofibers [6,7,11,12,13].

On the other hand, multi-walled carbon nanotubes (MWCNTs) are renowned for their exceptional electrical conductivity, mechanical strength, and thermal conductivity [14,15]. When integrated with SS-PCMs, MWCNTs can significantly enhance the thermal conductivity and electromagnetic interference (EMI) shielding effectiveness of the composite material [13,16,17,18,19,20,21,22,23]. Zhang et al. showed an enhancement in thermal conductivity with the introduction of carbon nanotubes as a filler in paraffin/expanded perlite [16]. Li et al. found that the thermal conductivity of montmorillonite/MWCNTs/paraffin composite is 34% greater than that of montmorillonite/paraffin composite and 65% higher than that of pure paraffin [17]. Mu et al. achieved a 29% increment of polyethylene/paraffin wax thermal conductivity by adding 3% MWCNTs [21]. Wu et al. also showed an improvement in the thermal conductivity of paraffin by adding graphene nanoplatelets (GNPs) and CNT nanoparticles [22]. Kholmanov et al. [13] showed that the effective thermal conductivity of Ultrathin Graphite Hybrid Foams composites can be further enhanced by growing extensive CNT networks directly from the graphite struts into the pore spaces. Hu et al. [23] created a continuous diamond–CNT hybrid by catalytically growing CNTs on the surface of a diamond film to encapsulate paraffin wax. This resulting composite demonstrated reduced supercooling, high thermal stability, and significantly improved thermal conductivity, greater than that of the original materials. Finally, Atinafu et al. presented a comparative study of biochar, activated carbon, expanded graphite and multi-walled carbon nanotubes for PCM encapsulation [24]. They found that the pore structure and surface functionality strongly affect the crystallinity of PCM and the heat storage capacity in the composite structures.

This work, for the first time, investigates the potential of three shape-stabilized phase change materials embedded in carbonaceous porous matrices combined with MWCNTs for thermal energy storage, while simultaneously providing EMI shielding. The study highlights the dual functionality of the composite material, effectively addressing both energy storage and EMI shielding within a single system. Octadecane was used as a phase change material and MWCNTs as a thermal and electrical conductivity enhancement additive. Three different carbonaceous matrices were used: (a) activated carbon derived from spent coffee; (b) red-mud carbon foam; and (c) expanded graphite. The presence of MWCNTs in the stabilized forms of PCMs is expected not only to improve their thermal properties but also to offer effective EMI shielding, making these composites suitable for advanced electronic and thermal management applications. This study aims to provide a comprehensive understanding of the material’s enhanced energy storage performance and EMI shielding effectiveness, thereby paving the way for future innovations in multifunctional composite materials.

## 2. Results and Discussion

**Figure 1:**
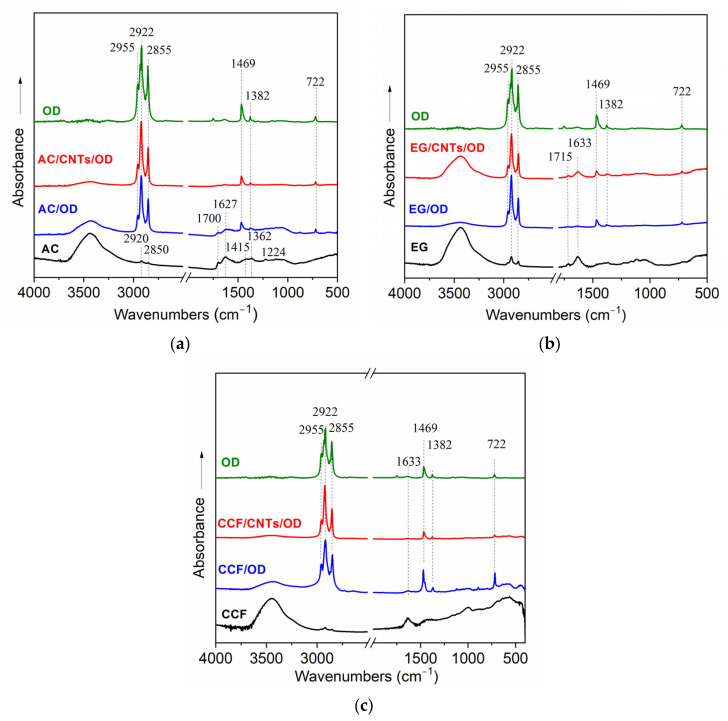
FT-IR spectra of: (**a**) activated carbon samples; (**b**) expanded graphite samples; and (**c**) ceramic carbon foam samples.

Figure 1 presents the FT-IR spectra of different carbon/OD and carbon/CNTs/OD composites, in comparison with the corresponding spectra of carbon matrices and raw OD.

The FT-IR spectra of activated carbon samples are shown in Figure 1a. The spectrum of the AC sample exhibits characteristic adsorption bands of activated carbon materials and confirms the presence of functional groups on its surface. More specifically, the broad adsorption band at 3440 cm^−1^ corresponds to O-H stretching vibrations probably derived from hydroxyl groups and/or water molecules [25]. The adsorption bands, observed at 2920 and 2850 cm^−1^, correspond to C-H stretching vibrations of aliphatic methylene groups and suggest the possible presence of residual organic content in the activated carbon [26]. Furthermore, the adsorption bands at 1700 and 1627 cm^−1^ are attributed to C=O vibrations of carboxylic groups and C=C stretching vibrations on aromatic groups, respectively [26,27], while they may also have contributions from vibrations of –COOH and/or –COO^−^ functional groups [27]. The small adsorption bands at 1415, 1362 and 1224 cm^−1^ are indicative of C-O stretching vibrations in carboxylic, ether, ester or hydroxyl groups, respectively [25,28]. The functional groups that are observed from the FT-IR spectra of AC contribute to its high surface reactivity and adsorption capacity. On the other hand, the spectra of AC/OD and AC/CNTs/OD composites exhibit additional features, compared with the spectrum of pure AC. The adsorption bands at 2920 and 2850 cm^−1^ appear obviously stronger and are similar to those bands observed on the octadecane spectrum indicating that they came from the C-H symmetric and asymmetric stretching vibrations of the methylene groups on the long alkane chain of octadecane [29]. Also, the new bands at 1469 and 722 cm^−1^, which are also shown on the OD spectrum, are attributed to C-H bending vibrations of aliphatic hydrocarbons and -CH_2_ group’s in-plane rocking vibrations of PCM molecules [30,31]. These features indicate the successful incorporation of PCM on the activated-carbon surface while similar results are also shown in the spectra of the expanded graphite and the ceramic carbon foam samples (Figure 1b and c, respectively).

**Figure 2:**
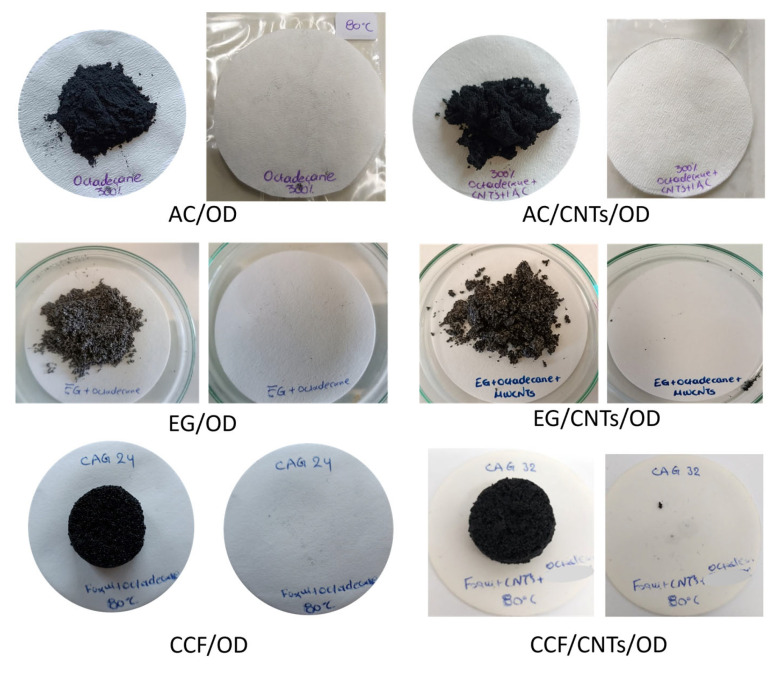
Photographs from filter paper after leaching tests at 80 °C for: activated carbon, expanded graphite and ceramic carbon foam samples.

Leaching tests for all composite materials were conducted, in order to evaluate the stability of the materials and Figure 2 shows the filter papers after conduction with the composite materials for 72 h at 80 °C. In all cases, the paper is clear that no oil stamps are observed indicating that no octadecane leakage occurred under these conditions. Thus, the leaching tests confirm that activated carbon, expanded graphite and ceramic carbon foam, with and without CNTs, effectively stabilize octadecane, offering promising solutions for applications requiring reliable thermal energy storage and minimal environmental impact.

**Figure 3:**
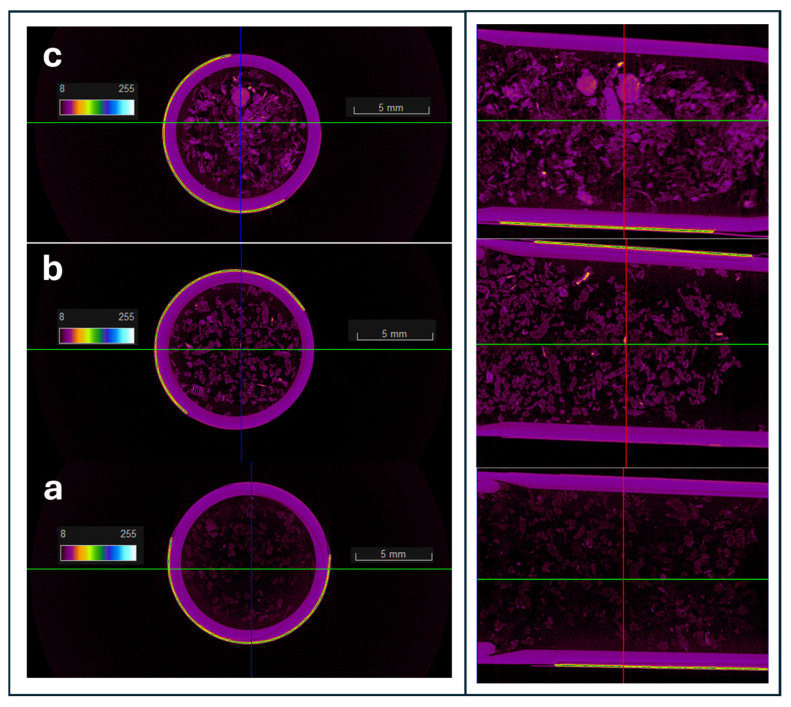
Microtomography CT images of: (**a**) EG; (**b**) EG/OD; and (**c**) EG/CNTs/OD. Green line is coronal (COR), blue line is sagittal (SAG) and red line is transverse (TRA) cross-sections of the samples.

The microtomography CT images of the three samples, EG, EG/OD and EG/CNTs/OD are shown in Figure 3a, b and c, respectively. According to Figure 3a, EG exhibits a highly porous structure, as indicated by the predominance of black areas in the image, which correspond to less dense regions filled with air voids. In the EG/OD composite, octadecane (indicated by purple areas) is uniformly distributed throughout the porous structure of EG. In the case of the EG/CNTs/OD composite, octadecane is also uniformly distributed, but some aggregates are observed, likely due to the presence of CNTs in these regions. The corresponding mirotomography CT images of the AC and the CCF samples are presented in Figures S1 and S2, respectively. The images of the pure samples of AC and CCF (Figures S1a and S2a, respectively) show similar features to those of the EG sample indicating also in these cases a highly porous structure consisting of low-density areas (black areas). The addition of OD to the porous matrices results in the images (Figures S1b and S2b) showing an increase in purple and green-yellow areas, indicating higher-density regions. These areas correspond to the uniform distribution of OD on both AC and CCF. For the AC/CNTs/OD composite, the CT microtomography image (Figure S1c) predominantly displays higher-density green-yellow areas, which is attributed to the presence of non-porous CNTs on the external surface of AC. In contrast, this effect is not observed in the CCF/CNTs/OD composite, suggesting that the CNTs are not distributed on the external surface of CCF. This is likely due to the macroporous structure of CCF, which may cause the CNTs to incorporate into the large pores instead.

**Figure 4:**
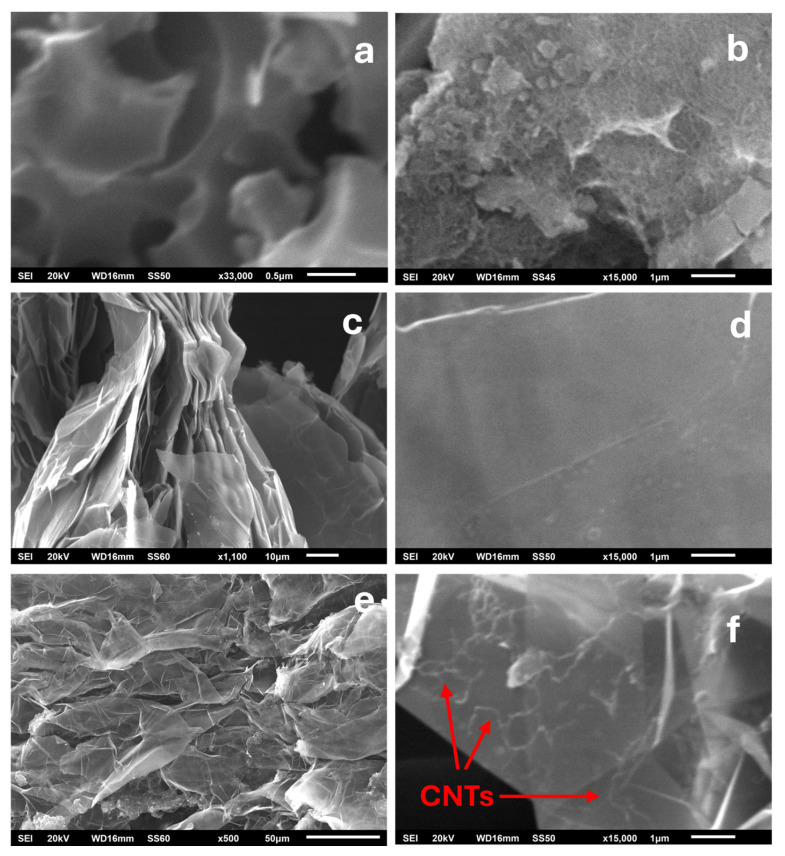
Scanning Electron Microscopy (SEM) images of: (**a**) AC matrix; (**b**) AC/CNTs/OD composite; (**c**,**d**) EG matrix; and (**e**,**f**) CCF/CNTs/OD composite.

In order to study the incorporation and distribution of CNTs on the carbon matrices, SEM images were collected and are presented in Figure 4. In the case of the AC/CNTs/OD sample (Figure 4b), the smooth and porous surface of AC (Figure 4a) is fully covered by CNTs which are uniformly dispersed on it and this finding is consistent with the microtomography CT images (Figure S1). Also, in the case of the EG/CNTs/OD sample (Figure 4e,f), CNTs are shown to be uniformly dispersed on the EG inert macroporous surface between its graphitic sheets. Finally, the SEM images of CCF (Figure S3a,b) reveal its macroporous structure, which probably accommodates the CNTs/OD mixture in its depth and it is not possible to distinguish carbon nanotubes with SEM in this particular structure, which is also consistent with the CT microtomography image (Figure S2) where the image of the CCF/CNTs/OD sample mainly consists of air voids. Overall, these findings from the SEM and CT microtomography analysis reveal distinct differences in the incorporation and distribution of CNTs across the various carbon matrices, suggesting that the interactions between CNTs and each carbon matrix are influenced by the distinct surface properties and porosity of the materials.

**Figure 5:**
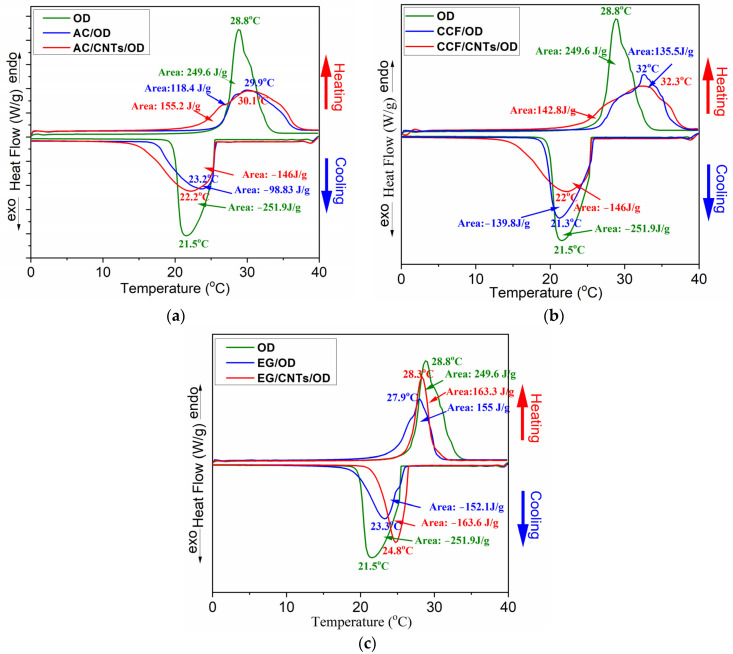
DSC curves for: (**a**) activated carbon samples; (**b**) ceramic carbon foam; and (**c**) expanded graphite samples.

**Figure 6:**
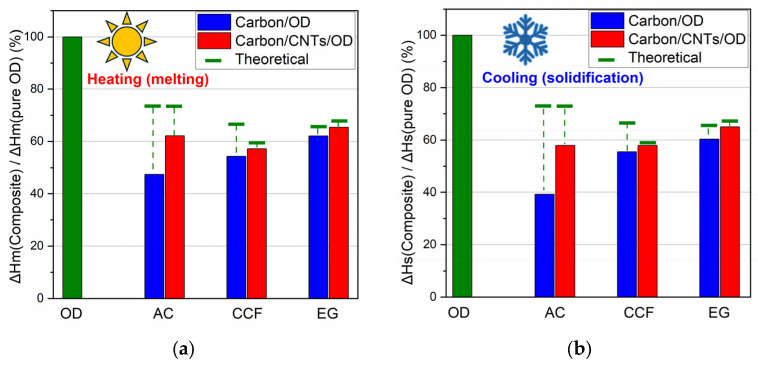
Comparative graphs for: (**a**) ΔHm% and (**b**) ΔHs% of the composites derived from DSC curves.

Differential Scaning Calorimetry (DSC) was used in order to investigate the ability of carbon/OD and carbon/CNTs/OD composites to store thermal energy and Figure 5 shows the DSC curves for all systems with the different carbon matrices. Table 1 summarizes the thermal properties derived from the DSC curves and Figure 6 shows comparative graphs for the ΔHm% and the ΔHs% of the composites according to each wt% OD loading. The expected theoretical values for ΔHm and ΔHs, according to OD loading, is also marked in this figure (green lines in Figure 6).

The DSC curve for pure octadecane exhibits an exothermic peak at 28.8 °C and an endothermic peak at 21.5 °C corresponding to its melting and solidification processes, respectively. The latent heat for melting (ΔHm) and solidification (ΔHs) is 249.6 and −251.9 J/g, respectively, which is inconsistent with the literature values for pure octadecane [32]. On the other hand, the DSC curves for the AC/OD and the AC/CNTs/OD composites (Figure 5a) show slightly shifted and broader endothermic and exothermic peaks compared to pure OD. The inclusion of carbon nanoparticles creates a heat conduction channel within the paraffin, potentially altering the mode of energy transfer in the paraffin [22]. The solicitation peaks slightly shifted to higher temperatures, compared to the pure OD. According to previous studies, this phenomenon is related to the presence of porous carbon, which facilitates the heterogeneous nucleation of the PCM, resulting in a reduced extent of supercooling in the composite PCMs and this is a crucial thermal property for the practical application of PCMs [24,33]. Additionally, the pore characteristics and surface tension can significantly influence the phase transition temperature of the synthesized composite PCMs [33]. Moreover, in the composite’s DSC curves, a reduction in ΔHs and ΔHm values is observed, reaching 39 and 47% of the corresponding values of pure OD (Table 1), which are lower than the expected theoretical values according to their PCM content (green lines in Figure 6). This also could happen due to the pore structure of AC carbon which limits the regular movement of the PCM chain [24].

Similar features are also shown in the DSC curves of the EG and the CCF composites (Figure 5b,c) and among the three carbon matrices without CNTs, the composite containing AC exhibited the lowest ΔH values and the greatest reduction from the theoretical ΔH values. The values for CCF show a smaller reduction, while the values for EG are very close to the theoretical ones. In addition, the DSC curves of the EG exhibit a similar shape to those of the pure PCM (they do not broaden as observed in the other two materials). These findings could be explained according to carbon matrice’s pore structure. The microporous structure significantly hinders the movement of PCM chains, the mesoporous structure to a lesser extent, and the macroporous structure even less. In the case of AC, which has mainly a microporous structure [26], OD molecules are probably impregnated into the micropores, while in the case of CCF and EG, which have larger meso and macropores, the OD molecules are arranged in the larger pores and the movement of the PCM chains is less obstructed.

The DSC curves of the carbon/CNTs/OD composites also show similar features. However, according to Table 1 and the comparative graphs for ΔHm% and ΔHs% (Figure 6a and b, respectively), the presence of CNTs seems to contribute to a lesser reduction in ΔH values compared to the composites with the pure carbon matrices. It is worth noting that half of the carbon in the composite materials was replaced with nanotubes (0.5 g carbon and 0.5 g CNTs in carbon/CNTs/OD composites instead of 1 g carbon in carbon/OD composites). In the case of the CNT composites, the ΔH values are closer to the calculated theoretical values according to each wt% OD loading. The presence of nanotubes enhances the thermal properties of octadecane probably due to the higher thermal conductivity of CNTs compared with AC, CCF and EG, facilitating more efficient heat transfer during the phase transitions of octadecane [22,34]. In addition, it seems that the paraffin loss observed in earlier studies can be prevented by the addition of CNTs [20].

**Figure 7:**
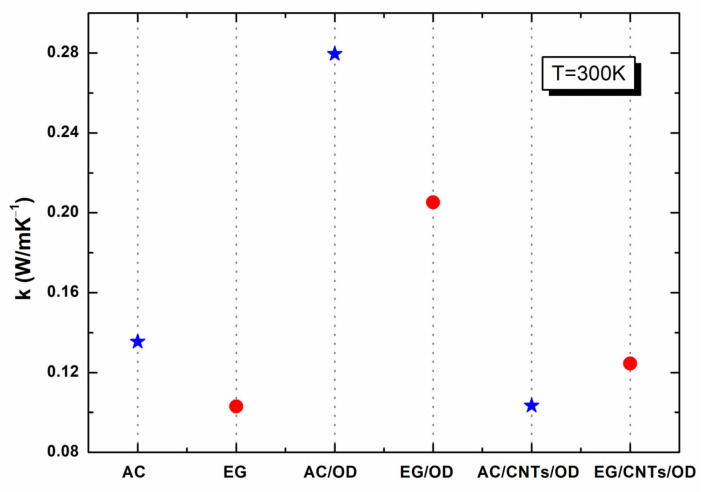
Thermal conductivity of studied samples (AC-based samples marked with blue stars, EG-based samples marked with red dots, at 300 K.

Figure 7 presents the thermal conductivity (TC) of the samples studied in this work at 300 K. As one can see from Figure 7, all samples exhibit low TC (0.10–0.28 W/m·K^−1^) resulting in inadequate heat transfer that prevents heat from penetrating into the PCM interior, thereby reducing their effectiveness in thermal energy storage (TES) applications [7]. The thermal conduction of AC and EG was measured as 0.13 and 0.10 W/m·K^−1^, respectively, while the corresponding values of AC/OD and EG/OD were 0.29 and 0.21 W/m·K^−1^, respectively. As already reported [35] and verified from our findings, AC, a porous form of graphite with a complex and imperfect structure, has a higher surface reactivity and adsorption capacity, which enhances its ability to store heat, verified by its higher TC compared to EG. Compared to OD (with a TC of ~0.153 W mK^−1^ in a liquid state [36]), the above-mentioned enhancement of both the AC/OD and the EG/OD samples in TC is mainly due to the many interconnected heat conduction channels of the carbon network structure, owing to the substitution of the air within the pores of AC (or EG) with OD [37], as already reported in the literature [38,39].

The incorporation of multi-walled carbon nanotubes (MWCNTs) into activated carbon or expanded graphite has garnered significant attention in materials science and engineering due to the unique properties that MWCNTs bring to composite materials. One of the critical properties affected by this addition is thermal conductivity, which is essential for various applications, including energy storage, thermal management, and filtration systems.

The addition of MWCNTs in activated carbon or expanded graphite combined with octadecane can lead to decreased overall thermal conductivity under specific conditions due to poor dispersion or interface effects that inhibit effective heat transfer pathways. As seen in Figure 7, the addition of MWCNTs in AC/OD or EG/OD leads to AC/CNTs/OD or EG/CNTs/OD samples with reduced TC values, equal to ~0.10 and ~0.13 W/m·K^−1^, respectively. This behavior could be attributed to insufficient formation of MWCNTs’ conductive pathways, poor dispersion leading to agglomeration, and ineffective interface interactions that hinder heat transfer, especially at low concentrations, where there may not be enough MWCNTs to create an effective network for heat conduction.

The EMI shielding (*SE*) efficiency of our samples *SE_T_* can be expressed as the sum of reflection (*SE_R_*), absorption (*SE_A_*) and multiple-reflection (*SE_M_*) as follows [40]:

*SE_T_*(dB) = *SE_R_*(dB) + *SE_A_*(dB) + *SE_M_*(dB) (1)

Generally, multiple-reflection at internal interfaces inside the material can be excluded if *SE* > 10–15 dB. Thus, we calculated the average *SE_T_* as

(2) $$SE=SE_{T}≜10\;\log_{10}\left(\frac{P_{inc}}{P_{trn}}\right)=10\;\log_{10}\left(\frac{1}{T}\right)=SE_{R}+SE_{A}$$

where

(3) $$SE_{R}=10\;\log_{10}\left(\frac{1}{1-R}\right)$$

(4) $$SE_{A}=10\;\log_{10}\left(\frac{1-R}{T}\right)$$

*SE_R_*, *SE_A_* refer to the reflection and absorption *SE*, respectively.

The higher the *SE* the better the shielding. The *SE* (also denoted as *SE_T_*, with *A*, *T*, *R* indicating the absorption, transmission and reflection, respectively) is usually quantified in terms of the logarithm of the incident power *P_inc_* over the transmitted power *P_trn_* [41,42] and thus expressed in decibels (dB).

**Figure 8:**
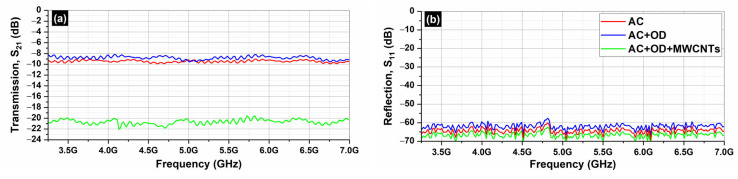
Transmission S_21_ (**a**) and reflection S_11_ (**b**) coefficients from 3.2 to 7.0 GHz (C-band) for AC samples.

Figure 8 depicts the S_21_ (transmission; Figure 8a) and the S_11_ parameters (reflection; Figure 8b) of the AC samples (AC, AC/OD, AC/CNTs/OD), in the frequency range 3.2–7.0 GHz.

**Figure 9:**
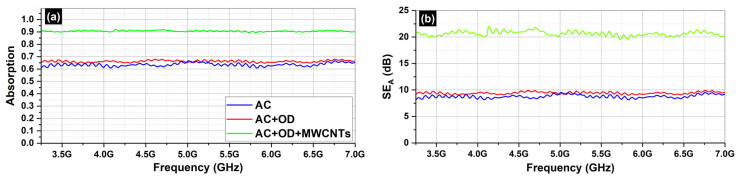
Absorption (**a**) and *SE_A_* (**b**) from 3.2 to 7.0 GHz (C-band) for AC samples.

Figure 9 depicts the absorption (Figure 9a) and the total shielding efficiency (*SE_T_*) (Figure 9b) spectra of the AC samples (AC, AC/OD, AC/CNTs/OD), in the frequency range 3.2–7.0 GHz.

**Figure 10:**
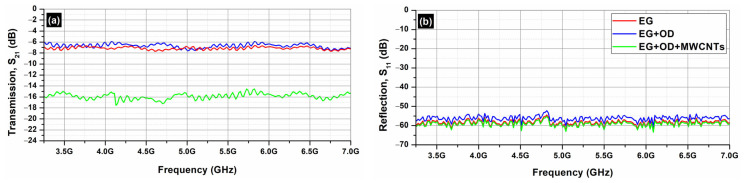
Transmission S_21_ (**a**) and reflection S_11_ (**b**) coefficients from 3.2 to 7.0 GHz (C-band) for EG samples.

Figure 10 depicts the S_21_ (transmission; Figure 10a) and the S_11_ parameters (reflection; Figure 10b) of the EG samples (EG, EG/OD, EG/CNTs/OD), in the frequency range 3.2–7.0 GHz. Figure 7 and Figure 9 clearly illustrate that the reflection of AC and EG samples is almost zero. As a result, the EMI shielding effect due to reflection (*SE_R_*) is also negligible and in all cases; the total SE is simply *SE_T_ = SE_A_* (see Equation (4)), which is in agreement with the literature on carbon-based composite materials [43].

**Figure 11:**
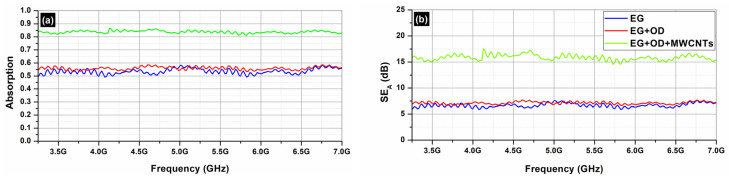
Absorption (**a**) and *SE_A_* (**b**) from 3.2 to 7.0 GHz (C-band) for EG samples.

Figure 11 depicts the absorption (Figure 11a) and the total shielding efficiency (*SE_T_*) (Figure 11b) spectra of the EG samples (EG, EG/OD, EG/CNTs/OD), in the frequency range 3.2–7.0 GHz.

As one can see, in all cases, the dominant shielding mechanism is absorption, which is also supported by the increasing absorption levels when MWCNTs are incorporated. As expected, and observed in Figure 8 and Figure 10, the composites’ electrical resistivity decreased with the addition of MWCNTs, and the composites’ EMI *SE* increased with the addition of MWCNTs. The decrease in electrical resistivity is due to an increase in the number of electrically conductive networks (due to MWCNTs), and the increase in EMI *SE* can be ascribed to the decrease in the composites’ electrical resistivity and the increase in absorption filler volume fraction [44].

On the other hand, as one can see from Figure 8 and Figure 10, all AC samples exhibit higher EMI *SE* levels compared to the corresponding EG samples. As already stated, the AC samples, a porous form of graphite with a complex and imperfect structure, have a higher surface reactivity and adsorption capacity [35] compared to EG ones. Indeed, pore size has a significant impact on electrical conductivity, as it affects the movement of charge carriers within the material. Expanded graphite primarily features larger pores, typically in the mesoporous, while activated carbon is known for its highly developed microporous structure, though it also contains mesopores and macropores. The presence of different pore sizes (microporous, mesoporous, macroporous) can create a complex network that enhances connectivity between conductive pathways, allowing for better transport pathways for charge carriers [45]. This is actually the reason that the AC samples exhibit better EMI *SE* properties compared to the EG ones.

**Figure 12:**
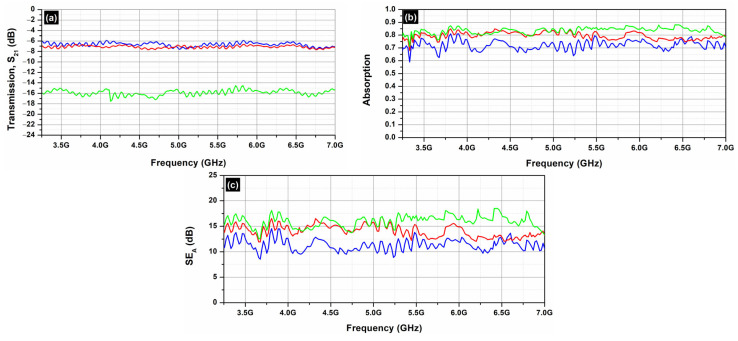
Transmission (**a**), absorption (**b**) and *SE_A_* (**c**) of CCF samples, from 3.2 to 7.0 GHz (C-band).

To continue with, the CCF samples were studied, as presented in Figure 12. CCF consists of 40% red mud. Red mud, a byproduct of the Bayer process used in aluminum extraction from bauxite ore, is primarily composed of iron oxides, aluminum oxides, silica, and other trace elements [46]. In principle, pure red mud has low electrical conductivity due to its high content of insulating materials like alumina, silica, etc. [47,48,49]. The addition of electrically insulating PCM does not contribute to the EMI shielding effectiveness of the samples, as seen in Figure 12c. As one can notice in Figure 12, even the addition of MWCNTs cannot significantly enhance the EMI shielding effectiveness of the samples, due to the low electrical conductivity of red mud.

As a result, in order to achieve effective *SE*, carbon-based materials are needed in order to achieve as high as possible electrical conductivity values.

## 3. Materials and Methods

### 3.1. Activated Carbon Production

The activated carbon production was held via microwave heating, replacing the conventional pyrolysis process and eliminating the experimental procedure at half of the time needed so far. More specifically, carbon was prepared from biomass materials, namely spent coffee with the help of a microwave reactor. Raw material-spent-coffee was first dried at 80 °C at least for 24 h to avoid humidity and after an amount of the product was mixed up with zinc chloride in 1:1 ratio. During the mixing procedure, distilled water was added and left at 80 °C for approximately 10 min. Afterwards, the produced sludge was placed into a microwave reactor and heated up until 400 °C with 700 mW power, under an inert atmosphere (argon flow of 0.5 mL/min). Subsequently, the produced activated carbon was poured into an HCl solution (1 N) and stirred for 24 h, and then the liquid was filtered and the precipitate was washed with deionized water to a neutral pH of approximately 7. Finally, the washed product was dried at 80 °C for 24 h.

### 3.2. Expanded Graphite Production

Expanded graphite (EG) was produced using commercially available expandable graphite (Sigma-Aldrich, Schnelldorf, Germany, sample code 808121). A 0.5 g sample of expandable graphite flakes was placed into a microwave oven for 90 s at 700 W power, with a supply of argon (Ar) gas flowing at 1 NL/min (NL: normal liters) to produce expanded graphite (EG). This process resulted in significant swelling to the desired extent (at least 30-fold), while notably, there was no substantial gas production or ignition inside the oven during the microwave exposure.

### 3.3. Preparation of Ceramic Carbon Foams (CCF) and Paraffin Encapsulation

The experimental procedure of Ceramic Carbon foams (CCF) as well as the hybrid foams-Octadecane (OD) synthesis are based on our previously published work [11]. Before the infiltration process, the CCFs were dried at 150 °C for at least two hours to eliminate any residual water. Subsequently, the dried CCFs were immersed in melted paraffins (n-octadecane (OD) from Sigma Aldrich). The paraffin was heated to 90 °C, and the foams were submerged for approximately five minutes to halt bubble extraction from the porous medium. The CCF compositions are listed in Table 2.

### 3.4. Activated Carbon-Expanded Graphite Paraffin Encapsulation

The paraffin encapsulation for activated carbon and expanded graphite was based on already published experimental routes [12,50]. Activated carbon and expanded graphite were first dried at 110 °C for at least 12 h in order to remove remaining water of the sample. Meanwhile, octadecane was heated above melting point, where was dissolved with absolute ethanol. The moment when the mixtures became lucid, activated carbon and expanded graphite were added. The solutions in both cases were stirred for 4 h under vigorous stirring. Finally, the mixtures were dried at 80 °C for at least 48 h. The activated carbon/octadecane—expanded graphite/octadecane ratio in both cases was 1:3. The accurate compositions are listed at Table 2.

### 3.5. Activated Carbon-Expanded Graphite Paraffin Encapsulation with Multiwalled Carbon Nanotubes

For the CNTs/OD composites incorporation into the pores of the shape stabilizer (AC/CNTs/OD and EG/CNTs/OD), the following experimental procedure was followed: 0.5 g of commercial multiwalled nanotubes (MWNTs) were placed in 42 mL of absolute ethanol, and then the mixture was placed into probe sonicator for half an hour (0.5 h) at 25 °C. Next, the mixture was placed at 80 °C to remove ethanol. At the same time, 1 g of the activated carbon and expanded graphite, respectively, were also placed in a furnace at 110 °C for 24 h in order to remove the naturally adsorbed water molecules from its surface. After drying the nanotubes, activated carbon and expanded graphite, 3 g of OD (the maximum loading rate was used) were placed in a beaker and heated to the melting point of the paraffin (Octadecane- OD, 28–30 °C), while 15 mL of absolute ethanol was then added dropwise to the melted paraffin, resulting in the creation of a blurred solution. After visual observation of the now homogenized (clear) solutions, 0.5 g of multi-walled nanotubes and 0.5 g of activated carbon and expanded graphite, respectively, were added. The mixtures were allowed to stir for four hours (4 h) at 600 rpm rate. The activated carbon/octadecane—expanded graphite/octadecane ratio in both cases was 1:3, while the exact compositions are listed in Table 2.

### 3.6. Ceramic Carbon Foams (CCF) Paraffin Encapsulation with Multiwalled Carbon Nanotubes

The development of the CCF/CNTs/OD composites was carried out by weighing 0.5 g of commercial multi-walled nanotubes (MWNTs) and then dispersing them in 50 mL of absolute ethanol. The heterogeneous mixture was placed in a probe sonicator for half an hour (0.5 h) at 25 °C. At the same time, OD paraffin was placed in a beaker and heated to its melting temperature. Then, the mixture with the nanotubes was added dropwise to the molten paraffin and the new heterogeneous mixture was placed in a water bath, at a temperature of 80 °C, where it remained for four hours (4 h) under continuous magnetic stirring. After this time period, the mixture was placed again in a water bath, this time at 20 °C, where it remained for one hour (1 h). After the one-hour pass, CCFs were dipped into the liquid mixture (OD with MWNTs) and then placed at 80 °C in a vacuum-drying oven. At this temperature, OD was melted once more and consequently, the mixture was encapsulated under vacuum into the CCFs. Finally, after the one-hour stay in the vacuum oven, the samples were removed from the mixture and left for at least twenty-four hours (24 h) at 80 °C on filter paper. This last stage was carried out in order to remove the excess paraffins that had not penetrated the pores, with continuous replacement of the papers, until the point at which leakage was no longer observed. The exact compositions are shown in Table 2.

### 3.7. Leaching Tests

In all cases, after the encapsulation process, leaching tests according to established standards were carried out to examine the ability of the composites to impregnate the OD. Originally, in all cases, the weight of the composites was measured, and subsequently, all the samples were placed onto filter papers at 30 °C for 8 h and then sequentially at 80 °C for 72 h. Finally, the weight was measured once more, and the filter paper was examined for possible leakages.

### 3.8. Infrared Spectroscopy

Infrared (FT-IR) spectroscopy was conducted on powdered samples dispersed in KBr pellets using a JASCO (Cremella, Italy) FT/IR-6000 Fourier transform spectrometer. The spectra were recorded as the average of 64 scans with a resolution of 4 cm^−1^, covering a wavenumber range of 400–4000 cm^−1^.

### 3.9. X-ray Computed Microtomography (Micro CT)

Micro-CT imaging was conducted using a Bruker SkyScan 1275 scanner (Billerica, MA, USA), featuring a distortion-free 3 Mp active flat-panel detector. The scans were performed with an accelerating voltage of 30 kV, a current of 50 μA, without the use of an aluminum or copper filter. The object distance was set to 20 mm, resulting in a pixel size of 15 μm. A 360° scan was completed with a 0.20° rotation step, taking approximately 25 min. The reconstruction process was carried out using Bruker’s NRecon—v2.1.0.1 software, while CTan v1.20.8.0 and Dataviewer 1.5.6.2 were employed for image analysis.

### 3.10. Scanning Electron Microscopy

Scanning Electron Microscopy (SEM) images were obtained using a JEOM JSM 6510-LV instrument Ltd. (Tokyo, Japan) equipped with an X-Act EDS-detector from Oxford Instruments, Abingdon, Oxfordshire, UK, (an acceleration voltage of 20 kV was applied).

### 3.11. Thermal Properties

Differential Scanning Calorimeter (DSC) was employed to analyze the thermal properties of the composite materials. All samples were subjected to a consistent heating and cooling rate of 10 K/min under a nitrogen (N_2_) atmosphere. The temperature ranged from 0 °C to 40 °C during heating and from 40 °C to 0 °C during cooling.

### 3.12. Thermal Conductivity

A commercially available measurement device was used, (Trident Thermal Conductivity Instrument, C-Therm Technologies Ltd., Fredericton, NB, Canada), in which the so-called Modified Transient Plane Source (MTPS) method is employed. According to the method, a spiral metallic element is used as heater. The element is covered by a polymeric film, so it is electrically insulated but does not thermally conduct. A constant current is applied to the element, and a subsequent amount of heat evolves. Around the heating element, there is a guard ring that prevents the in-plane heat transport, thus the produced heat is transferred vertically, with respect to the spiral plane. Since the element exhibits a finite resistance, the produced heat is proportional to the voltage applied across the element. Apparently, the produced heat leads to a change in the temperature of the element, and such a change can be recorded by the element itself, which can be used as a temperature sensor as well. Therefore, the temperature change is related to the voltage across the heater/sensor. By attaching a material above the element, a rise in the temperature at the interface between the sample and the sensor occurs, which induces a change in the voltage drop across the heater. The rate of increase in the sensor voltage is used to determine the thermal properties of the sample. The voltage is factory-calibrated to temperature. The thermal conductivity is inversely proportional to the rate of increase in the temperature at the point of contact between the sensor and the sample.

Each sample was measured several times, and the measured values were directly extracted by the C-Therm v 3.6.1.57 software. Finally, it must be noted that before measuring the samples of interest, the device was calibrated against standard materials, as proposed by the manufacturer.

### 3.13. Electromagnetic Interference (EMI) Shielding Properties

The EMI shielding effectiveness properties were evaluated using a P9372A Keysight Streamline Vector Network Analyzer (Keysight, Santa Rosa, CA, USA) and two sets of microwave standard 15 dB gain waveguides (WR 187 and WR 147, respectively, obtained from Advanced Technical Materials Inc. (ATM, Patchogue, NY, USA) covering a broad frequency band in the range of 3.2–7.0 GHz (C-band), which is used for long-distance radio telecommunications, such as satellite communications transmissions, Wi-Fi devices, cordless telephones, weather radar systems, etc. In particular, every sample was placed in the middle of each set of waveguides, and its scattering parameters (S-parameters; S_11_, S_12_, S_22_, S_21_) were recorded.

## 4. Conclusions

In conclusion, this study investigated the development and performance of carbonaceous shape-stabilized octadecane/MWCNTs composite materials, focusing on activated carbon, ceramic carbon foam, and expanded graphite as the support matrices. The findings highlight the potential of these composites for enhanced energy storage and electromagnetic interference (EMI) shielding applications.

SEM analysis, supported by CT microtomography, revealed that CNTs are uniformly dispersed on both the AC and the EG matrices, with the AC surface being completely covered by CNTs. However, in the case of CCF, the macroporous structure likely encapsulates the CNTs/OD mixture within its depth, making them indistinguishable in SEM images. These observations highlight the varying interactions between CNTs and different carbon matrices, influenced by the inherent surface properties and porosity of each material.

The leaching tests affirmed that all three carbon matrices—activated carbon, expanded graphite, and ceramic carbon foam—effectively stabilize octadecane, with or without MWCNTs. This stability indicates their suitability for applications that require dependable thermal energy storage while maintaining minimal environmental impact.

Among the matrices without MWCNTs, the activated carbon composites showed the lowest ΔH values and the most significant reduction from theoretical ΔH values. Ceramic carbon foam displayed a smaller reduction in ΔH values, while expanded graphite values remained close to theoretical expectations. The introduction of MWCNTs markedly enhanced the ΔH values across all matrices, resulting in more efficient energy storage.

In terms of EMI shielding, the primary mechanism observed was absorption, which was further enhanced by the incorporation of MWCNTs in the case of the AC and the EG samples. Notably, the EMI shielding effectiveness of the activated carbon samples was superior to that of the expanded graphite samples. On the other hand, in the case of the CCF samples, the EMI shielding effectiveness of the samples was not significantly enhanced, due to the low electrical conductivity of red mud.

These findings open up new possibilities for advanced materials that can meet the rigorous demands of modern energy storage systems and EMI protection technologies.

## Figures and Tables

**Table 1:** Melting and solidification temperatures and enthalpies.

	Ts	Tm	ΔHs	ΔHm	OD ΔHs(%)	OD ΔHm(%)	g OD/g Carbon	OD %w.t.
OD	21.5	28.8	−251.9	249.6	100	100	--	100
AC/OD	23.2	29.9	−98.83	118.4	39.23	47.44	2.738	73
AC/CNTs/OD	22.2	30.1	−146.0	155.2	57.96	62.18	2.743	73
CCF/OD	21.3	32	−139.8	135.5	55.49	54.29	1.966	66
CCF/CNTs/OD	22.0	32.3	−146.0	142.8	57.96	57.21	1.345	58
EG/OD	23.3	27.9	−152.0	155	60.34	62.10	1.914	65
EG/CNTs/OD	24.8	28.3	−163.6	163.3	64.95	65.42	1.990	67

**Table 2:** Compositions of carbon/OD and carbon/CNTs/OD composites before and after encapsulation process.

Sample	Initial Weight (g)	Initial Weight-OD (g)	Initial Weight-OD-80 °C (g)	OD Loading (w.t.%)
AC/OD	1.0	4.0	3.738	73
AC/CNTs/OD	1.0	4.0	3.743	73
CCF/OD	2.143	6.785	6.357	66
CCF/CNTs/OD	2.053	4.9276	4.8146	58
EG/OD	1.0	4.0	2.914	66
EG/CNTs/OD	1.0	4.0	2.990	67

## Data Availability

The data presented in this study are available upon request from the corresponding author.

## Supplementary Materials

The following supporting information can be downloaded at: https://www.mdpi.com/article/10.3390/molecules29184363/s1, Figure S1: Microtomography CT images of (a) AC, (b) C/OD and (c) AC/CNTs/OD. Figure S2: Microtomography CT images of (a) CCF, (b) CCF/OD and (c) CCF/CNTs/OD.

## References

[1] Faraj K., Khaled M., Faraj J., Hachem F., Castelain C. (2021). A Review on Phase Change Materials for Thermal Energy Storage in Buildings: Heating and Hybrid Applications. J. Energy Storage.

[2] Zachariah S.M., Antony T., Grohens Y., Thomas S. (2022). From Waste to Wealth: A Critical Review on Advanced Materials for EMI Shielding. J. Appl. Polym. Sci..

[3] Kasaeian A., Pourfayaz F., Khodabandeh E., Yan W.M. (2017). Experimental Studies on the Applications of PCMs and Nano-PCMs in Buildings: A Critical Review. Energy Build..

[4] Liu X., Yang F., Li M., Sun C., Wu Y. (2022). Development of Cost-Effective PCM-Carbon Foam Composites for Thermal Energy Storage. Energy Rep..

[5] Chakraborty A., Noh J., Mach R., Shamberger P., Yu C. (2022). Thermal Energy Storage Composites with Preformed Expanded Graphite Matrix and Paraffin Wax for Long-Term Cycling Stability and Tailored Thermal Properties. J. Energy Storage.

[6] Motahar S., Alemrajabi A.A., Khodabandeh R. (2015). Enhanced Thermal Conductivity of N-Octadecane Containing Carbon-Based Nanomaterials. Heat Mass Transf..

[7] Chen X., Cheng P., Tang Z., Xu X., Gao H., Wang G. (2021). Carbon-Based Composite Phase Change Materials for Thermal Energy Storage, Transfer, and Conversion. Adv. Sci..

[8] Atinafu D., Choi J., Yun B.Y., Nam J., Kim H., Kim S. (2023). Energy Storage and Key Derives of Octadecane Thermal Stability during Phase Change Assembly with Animal Manure-Derived Biochar. Environ. Res..

[9] Rathore P.K.S., Shukla S.K. (2021). Enhanced Thermophysical Properties of Organic PCM through Shape Stabilization for Thermal Energy Storage in Buildings: A State of the Art Review. Energy Build..

[10] Abdeali G., Bahramian A.R., Abdollahi M. (2020). Review on Nanostructure Supporting Material Strategies in Shape-Stabilized Phase Change Materials. J. Energy Storage.

[11] Gioti C., Karakassides A., Asimakopoulos G., Baikousi M., Salmas C.E., Viskadourakis Z., Kenanakis G., Karakassides M.A. (2022). Multifunctional Carbon-Based Hybrid Foams for Shape-Stabilization of Phase Change Materials, Thermal Energy Storage, and Electromagnetic Interference Shielding Functions. Micro.

[12] Gioti C., Vasilopoulos K.C., Baikousi M., Salmas C.E., Ntaflos A., Paipetis A.S., Viskadourakis Z., Ikram R., Agathopoulos S., Kenanakis G. (2024). Enhanced Gypsum Boards with Activated Carbon Composites and Phase Change Materials for Advanced Thermal Energy Storage and Electromagnetic Interference Shielding Properties. Micro.

[13] Kholmanov I., Kim J., Ou E., Ruoff R.S., Shi L. (2015). Continuous Carbon Nanotube-Ultrathin Graphite Hybrid Foams for Increased Thermal Conductivity and Suppressed Subcooling in Composite Phase Change Materials. ACS Nano.

[14] Kukovecz A., Kozma G., Kónya Z. (2013). Multi-Walled Carbon Nanotubes. Springer Handbook of Nanomaterials.

[15] Yang D., Zhang Q., Chen G., Yoon S., Ahn J., Wang S., Zhou Q., Wang Q., Li J. (2002). Thermal Conductivity of Multiwalled Carbon Nanotubes. Phys. Rev. B.

[16] Zhang X., Wen R., Huang Z.-H., Tang C., Huang Y., Liu Y., Fang M., Wu X., Min X., Xu Y. (2017). Enhancement of Thermal Conductivity by the Introduction of Carbon Nanotubes as a Filler in Paraffin/Expanded Perlite Form-Stable Phase-Change Materials. Energy Build..

[17] Li M., Guo Q., Nutt S. (2017). Carbon Nanotube/Paraffin/Montmorillonite Composite Phase Change Material for Thermal Energy Storage. Sol. Energy.

[18] Zhou L., Wang X., Wu Q., Ni Z., Zhou K., Wen C., Yan X., Xie T. (2024). Carbon Nanotube Sponge Encapsulated Ag-MWCNTs/PW Composite Phase Change Materials with Enhanced Thermal Conductivity, High Solar-/Electric-Thermal Energy Conversion and Storage. J. Energy Storage.

[19] Wang M., Wu Z., Liu A., Wang Y., Xie H. (2022). Carbon Nanotube/Nickel Foam-Mannitol Phase Change Composite Material for Medium-Temperature Solar Energy Storage and Conversion. J. Energy Storage.

[20] Poikelispää M., Honkanen M., Vippola M., Sarlin E. (2020). Effect of Carbon Nanotubes and Nanodiamonds on the Heat Storage Ability of Natural Rubber Composites. J. Elastomers Plast..

[21] Mu M., McNally T. (2022). The Effect of Multi-Walled Carbon Nanotubes on the Thermo-Physical Properties of Shape Stabilised Phase Change Materials for Buildings Based on High Density Polyethylene and Paraffin Wax. J. Energy Storage.

[22] Wu X., Gao M., Wang K., Wang Q., Cheng C., Zhu Y., Zhang F., Zhang Q. (2021). Experimental Study of the Thermal Properties of a Homogeneous Dispersion System of a Paraffin-Based Composite Phase Change Materials. J. Energy Storage.

[23] Hu N., Haichao L., Wei Q., Zhou K., Zhu W., Zhang L., Li S., Ye W., Jiao Z., Luo J. (2020). Continuous Diamond-Carbon Nanotube Foams as Rapid Heat Conduction Channels in Composite Phase Change Materials Based on the Stable Hierarchical Structure. Compos. Part B Eng..

[24] Atinafu D.G., Yun B.Y., Wi S., Kang Y., Kim S. (2021). A Comparative Analysis of Biochar, Activated Carbon, Expanded Graphite, and Multi-Walled Carbon Nanotubes with Respect to PCM Loading and Energy-Storage Capacities. Environ. Res..

[25] Dandekar A., Baker R.T.K., Vannice M.A. (1998). Characterization of Activated Carbon, Graphitized Carbon Fibers and Synthetic Diamond Powder Using TPD and DRIFTS. Carbon.

[26] Asimakopoulos G., Baikousi M., Kostas V., Papantoniou M., Bourlinos A.B., Zbořil R., Karakassides M.A., Salmas C.E. (2020). Nanoporous Activated Carbon Derived via Pyrolysis Process of Spent Coffee: Structural Characterization. Investigation of Its Use for Hexavalent Chromium Removal. Appl. Sci..

[27] Baikousi M., Georgiou Y., Daikopoulos C., Bourlinos A.B., Filip J., Zbořil R., Deligiannakis Y., Karakassides M.A. (2015). Synthesis and Characterization of Robust Zero Valent Iron/Mesoporous Carbon Composites and Their Applications in Arsenic Removal. Carbon.

[28] Fanning P.E., Vannice M.A. (1993). A DRIFTS Study of the Formation of Surface Groups on Carbon by Oxidation. Carbon.

[29] Woo H.Y., Lee D., Yoon T., Kim J., Chae J.-Y., Paik T. (2021). Sub-100-Nm Nearly Monodisperse n-Paraffin/PMMA Phase Change Nanobeads. Nanomaterials.

[30] Zhuang X., Zhang Y., Cai C., Zhang J., Zhu Y. (2018). Design the Magnetic Microencapsulated Phase Change Materials with Poly(MMA-MAA) @ n-Octadecane Modified by Fe_3_O_4_. Sci. Rep..

[31] Zhang K., Wang J., Xu L., Xie H., Guo Z. (2021). Preparation and Thermal Characterization of N-Octadecane/Pentafluorostyrene Nanocapsules for Phase-Change Energy Storage. J. Energy Storage.

[32] Faden M., Höhlein S., Wanner J., König-Haagen A., Brüggemann D. (2019). Review of Thermophysical Property Data of Octadecane for Phase-Change Studies. Materials.

[33] Atinafu D.G., Dong W., Wang C., Wang G. (2018). Synthesis of Porous Carbon from Cotton Using an Mg(OH)_2_ Template for Form-Stabilized Phase Change Materials with High Encapsulation Capacity, Transition Enthalpy and Reliability. J. Mater. Chem. A.

[34] Xu C., Zhang H., Fang G. (2022). Review on Thermal Conductivity Improvement of Phase Change Materials with Enhanced Additives for Thermal Energy Storage. J. Energy Storage.

[35] Hasumi S., Iwakami S., Sasaki Y., Faraezi S., KHAN M., Ohba T. (2023). Fast Ion Transfer Associated with Dehydration and Modulation of Hydration Structure in Electric Double-Layer Capacitors Using Molecular Dynamics Simulations and Experiments. Batteries.

[36] Yu S., Wang X., Wu D. (2014). Microencapsulation of N-Octadecane Phase Change Material with Calcium Carbonate Shell for Enhancement of Thermal Conductivity and Serving Durability: Synthesis, Microstructure, and Performance Evaluation. Appl. Energy.

[37] Hekimoğlu G., Sarı A., Gencel O., Tyagi V.V., Sharma R.K. (2023). Activated Carbon/Expanded Graphite Hybrid Structure for Development of Nonadecane Based Composite PCM with Excellent Shape Stability, Enhanced Thermal Conductivity and Heat Charging-Discharging Performance. Therm. Sci. Eng. Prog..

[38] Yang H., Wang Y., Yu Q., Cao G., Sun X., Yang R., Zhang Q., Liu F., Di X., Li J. (2018). Low-Cost, Three-Dimension, High Thermal Conductivity, Carbonized Wood-Based Composite Phase Change Materials for Thermal Energy Storage. Energy.

[39] Zhang W., Zhang X., Zhang X., Yin Z., Liu Y., Fang M., Wu X., Min X., Huang Z. (2019). Lauric-Stearic Acid Eutectic Mixture/Carbonized Biomass Waste Corn Cob Composite Phase Change Materials: Preparation and Thermal Characterization. Thermochim. Acta.

[40] Verma M., Singh A.P., Sambyal P., Singh B.P., Dhawan S.K., Choudhary V. (2014). Barium Ferrite Decorated Reduced Graphene Oxide Nanocomposite for Effective Electromagnetic Interference Shielding. Phys. Chem. Chem. Phys..

[41] Al-Saleh M.H., Saadeh W.H., Sundararaj U. (2013). EMI Shielding Effectiveness of Carbon Based Nanostructured Polymeric Materials: A Comparative Study. Carbon.

[42] Viskadourakis Z., Vasilopoulos K.C., Economou E.N., Soukoulis C.M., Kenanakis G. (2017). Electromagnetic Shielding Effectiveness of 3D Printed Polymer Composites. Appl. Phys. Mater. Sci. Process..

[43] Zheng X., Hu Q., Wang Z., Nie W., Wang P., Li C. (2021). Roll-to-Roll Layer-by-Layer Assembly Bark-Shaped Carbon Nanotube/Ti3C2Tx MXene Textiles for Wearable Electronics. J. Colloid Interface Sci..

[44] Al-Saleh M., Sundararaj U. (2009). Electromagnetic Interference Shielding Mechanisms of CNT/Polymer Composites. Carbon.

[45] Khamkeaw A., Asavamongkolkul T., Perngyai T., Jongsomjit B., Phisalaphong M. (2020). Interconnected Micro, Meso, and Macro Porous Activated Carbon from Bacterial Nanocellulose for Superior Adsorption Properties and Effective Catalytic Performance. Molecules.

[46] Silveira N.C.G., Martins M.L.F., Bezerra A.C.S., Araújo F.G.S. (2021). Red Mud from the Aluminium Industry: Production, Characteristics, and Alternative Applications in Construction Materials—A Review. Sustainability.

[47] Sverdrup H.U., Ragnarsdottir K.V., Koca D. (2015). Aluminium for the Future: Modelling the Global Production, Market Supply, Demand, Price and Long Term Development of the Global Reserves. Resour. Conserv. Recycl..

[48] Mondillo N., Herrington R., Boni M., Alderton D., Elias S.A. (2021). Bauxites. Encyclopedia of Geology.

[49] Ruys A. (2019). Bauxite: The Principal Aluminum Ore. Alumina Ceramics.

[50] Khadiran T., Hussein M.Z., Zainal Z., Rusli R. (2015). Shape-Stabilised *n*-Octadecane/Activated Carbon Nanocomposite Phase Change Material for Thermal Energy Storage. J. Taiwan Inst. Chem. Eng..

