# Supplementary material for: Carbonaceous Shape-Stabilized Octadecane/Multi-Walled Carbon Nanotube Composite Materials for Enhanced Energy Storage and Electromagnetic Interference Shielding
Source: Molecules. 2024 Sep 13;29(18):4363. doi: 10.3390/molecules29184363 (PMC11434077; doi:10.3390/molecules29184363)
Supplement: Supplementary file 1 [file molecules-29-04363-s001.zip › molecules-3161403-supplementary.pdf]

Carbonaceous shape stabilized octadecane/ Multi-Walled Carbon Nanotubes composite materials for enhanced energy storage and electromagnetic interference shielding

Maria Baikousi [1,*], Christina Gioti [1], Konstantinos C. Vasilopoulos [1], Argyri Drymiskianaki [2,3], Vassilis M. Papadakis [2,4], Zacharias Viskadourakis [2], Angelos Ntaflos [1], Dimitrios Moschovas [1], Alkiviadis S. Paipetis [1], George Kenanakis [2] and Michael A. Karakassides [1,*]

Supporting Information

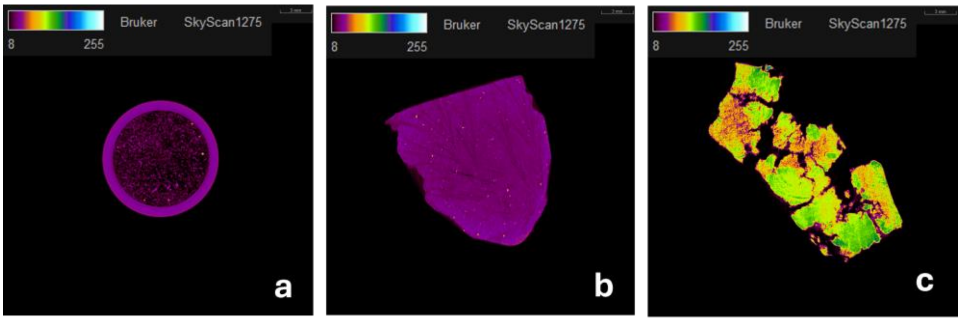

Figure S1. Microtomography CT images of a) AC, b) C/OD and c) AC/CNTs/OD.

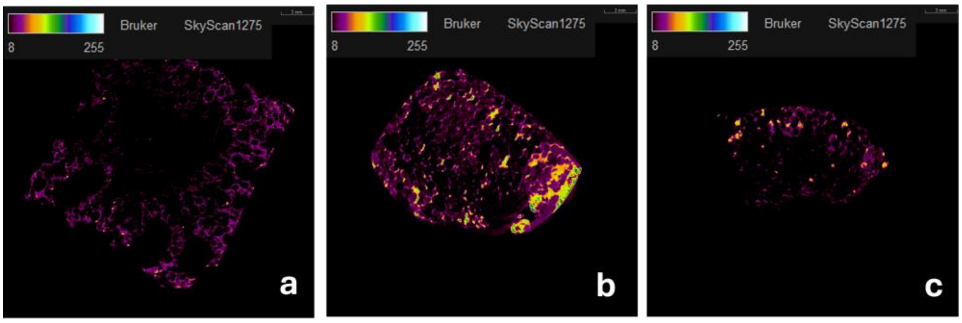

Figure S2. Microtomography CT images of a) CCF, b) CCF/OD and c) CCF/CNTs/OD

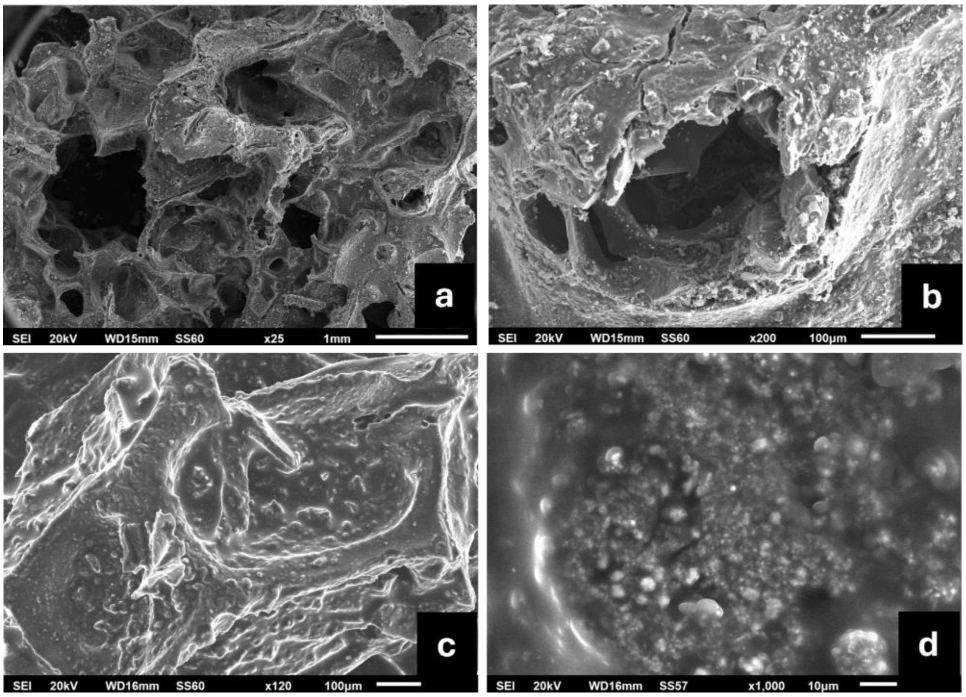

Figure S3. Scanning Electron Microscopy (SEM) images of a) CCF matrix (a and b) and CCF/CNTs/OD composite (c and d).
